# The role of injections of mesenchymal stem cells as an augmentation tool in rotator cuff repair: a systematic review

**DOI:** 10.1016/j.xrrt.2024.12.003

**Published:** 2025-01-13

**Authors:** Nuno Vieira Ferreira, Renato Andrade, Tânia Pinto Freitas, Clara de Campos Azevedo, João Espregueira-Mendes, António J. Salgado, Nuno Sevivas

**Affiliations:** a Life and Health Sciences Research Institute (ICVS), School of Health Sciences, University of Minho, Braga, Portugal; b ICVS/3B’s, PT Government Associate Laboratory, Braga/Guimarães, Portugal; c Hospital de Santa Maria Maior Barcelos, Barcelos, Portugal; d Hospital dos Lusíadas Braga, Braga, Portugal; e Instituto de Investigação em Ortopedia e Medicina Desportiva, Braga, Portugal; f Clínica Espregueira - FIFA Medical Centre of Excellence, Porto, Portugal; g Dom Henrique Research Centre, Porto, Portugal; h Porto Biomechanics Laboratory (LABIOMEP), University of Porto, Porto, Portugal; i Hospital dos SAMS de Lisboa, Lisbon, Portugal; j Hospital CUF Tejo, Lisbon, Portugal; k School of Medicine, University of Minho, Braga, Portugal; l 3B’s Research Group – Biomaterials, Biodegradables and Biomimetics, University of Minho, Headquarters of the European Institute of Excellence on Tissue Engineering and Regenerative Medicine, Guimarães, Portugal; m Trofa Saúde Group, Vila do Conde, Portugal; n Centro Hospitalar Médio Ave, Famalicão, Portugal

**Keywords:** Rotator cuff, Tear, Repair, Mesenchymal stem cells, Healing, Injection, Augmentation

## Abstract

**Background:**

Arthroscopic repair is currently the gold standard for the surgical treatment of rotator cuff tears, but the retear rates remain unacceptably high. Mesenchymal stem cells (MSCs) may play a role in the local biology and enhance tendon-to-bone healing during rotator cuff repair. However, the scientific literature is still not well systematized on the effects of injection of MSCs as an augmentation tool for rotator cuff repair. Our goal was to investigate the effect of injections of MSCs to augment rotator cuff repair in patients with rotator cuff tear.

**Methods:**

PubMed and EMBASE were searched up to June 2022 for clinical studies that applied MSCs injections to augment rotator cuff repair. Imaging, patient-reported outcomes measures, shoulder range of motion and strength were collected. Quantitative synthesis included within- and between-group mean differences with the within-group percentage of minimal clinically important difference for each study and continuous outcomes, and relative risks (RR) for retears and adverse events. Quantitative synthesis was computed with 95% confidence intervals (CIs).

**Results:**

We included 5 studies comprising a total of 228 individuals with a weighted mean age of 59.3 ± 1.2 years. Three studies used bone marrow MSCs and two studies applied adipose-derived MSCs. Patient-reported outcomes measures, shoulder range of motion, and strength improved significantly in all MSCs groups, with minimal clinically important differences ranging from 120% to 679% of established cut-off. When compared to rotator cuff repair alone, the MSCs groups did not result in improved outcomes. The MSCs group showed significant protective effect at the mid-term (RR = 0.52, 95% CI 0.27-0.98) and long-term (RR = 0.24, 95% CI 0.11-0.53).

**Conclusion:**

There are no differences in clinical and functional outcomes between rotator cuff repair with or without augmentation with MSCs. However, there may be a protective effect against retear at the mid-term and long-term follow-up when augmenting the repair with MSCs. The literature on this topic is still preliminary and the quality and certainty of evidence is limited.

Arthroscopic repair is currently the gold standard for the surgical treatment of rotator cuff tears^2^^,^^6^^,^^49^^,^^77^^,^^89^^,^^114^ and results in a high rate of successful objective outcomes in more than 85% of the patients.^13^^,^^14^^,^^117^ The rate of tendon healing is reported to be 80% for small tears, but it declines to about 30% for large/massive tears^8^ with a retear rate that can go as high as 94%,^10^^,^^26^^,^^44^^,^^100^^,^^120^ thus decreasing the positive clinical outcomes at long-term follow-up.^35^^,^^99^^,^^117^

Healing the tendon back to the bone depends on mechanical factors -biomechanically stronger fixation techniques improve tendon healing to the bone^1^^,^^20^^,^^22^^,^^38^^,^^62^ – as well as on biological factors.^9^^,^^28^^,^^59^ The biologic failure of the tendon-bone interface healing is mostly related to muscle-tendinous degeneration, reduced cellular activity, and decreased tendon healing potential.^59^^,^^65^ Cell-based therapies have recently received increasing interest due to their potential to promote tendon healing. Adult mesenchymal stem cells (MSCs) have emerged as a promising therapeutic option in the regenerative medicine of different musculoskeletal tissues, including the tendon.^33^^,^^76^

The rationale of the use of MSCs-based therapies in rotator cuff repair is to facilitate rotator cuff healing by differentiating into tenocytes or osteoblasts, by recruiting and stimulating progenitors, and by reducing inflammation.^75^ Patients with rotator cuff tears also demonstrate decreased number of resident MSCs,^40^ highlighting the potential of supplementing the rotator cuff repair with exogenous MSCs.^75^

Up until now, there have been several preclinical animal studies on the potential of MSCs and their derivatives for the repair of rotator cuff tears.^18^^,^^25^^,^^29^, ^30^, ^31^, ^32^, ^33^^-^^34^^,^^50^^,^^52^^,^^76^^,^^85^^,^^86^^,^^90^^,^^91^^,^^102^^,^^106^^,^^108^^,^^111^^,^^112^ However, the evidence of the use of MSCs and their derivates as an augmentation to rotator cuff repair in humans is not systematized and summarized in the literature. Our goal was to conduct a systematic review to investigate the effect of injections of MSCs and their derivates to augment rotator cuff repair in patients with rotator cuff tears. We hypothesized that rotator cuff repair augmented with injections of MSCs improves clinical results and decreased the risk for tendon retear as compared to rotator cuff repair alone.

## Materials and methods

This systematic review was conducted according to the Preferred Reporting Items for Systematic Reviews and Meta-Analyses 2020 statement.^79^ The protocol for this systematic review was registered a priori at International Prospective Register of Systematic Reviews with the number CRD42020122123.

### Eligibility criteria

The eligibility criteria were set according to the Participants, Intervention, Comparison, Outcome, and Study design framework. As target population (P), we included studies that were comprised by adult individuals of either sex with rotator cuff tears. Revision cases of rotator cuff retears were excluded. Participants with concomitant pathologies, such as osteoarthritis or diabetes were also excluded. Studies comprising mixed populations that do not provide separate data for participants with rotator cuff tears were excluded. The included intervention (I) comprised rotator cuff surgical repair when supplemented with injections of MSCs and/or their derivatives (secretome); studies reporting the results of MSCs as component of a scaffold or patch, or associated to a concomitant bone marrow-stimulating techniques (*e*.*g*., microfracture) were excluded. For being eligible, MSCs must have been isolated from donor sources and injected locally during the rotator cuff repair procedure. We included all sources of MSCs, either autogenic or allogenic, and harvested from bone marrow, adipose, synovium, umbilical cord, placenta, tendon or bursa. As comparator group (C), we considered surgical repair of the rotator cuff that was not augmented with MSCs and/or their derivatives. As outcomes (O), we considered all patient-reported outcome measures (PROMs) for pain, improvement, satisfaction and function. Other outcomes of interest included enthesis quality, retear rate, and shoulder biomechanical function, such as range of motion. The study design (S) that was eligible for inclusion was randomized clinical trials, prospective or retrospective cohort studies and case-control studies. Due to the limited research in this field, we also considered case series for inclusion. However, a minimum of 10 participants was required for inclusion. Conference abstracts were not considered for inclusion.

### Search strategy

Literature searches were conducted on PubMed and EMBASE databases. Searches were performed independently by two authors (NVF and NS) from database inception up to June 30, 2022. The full search strategy for each database is described on Supplement 1. The reference list of included studies and other relevant reviews was scanned for other potential eligible studies.

### Study selection

All records were exported to EndNote X7 (Thomson and Reuters, Philadelphia, PA, USA) and the duplicate records were removed using the software automated tool and hand-checked for any missing duplicate records. Two authors (NVF and NS) scanned independently all titles and abstracts and identified all potentially relevant studies that required further analysis. The full-text of each of the potentially relevant studies was analyzed according to the eligibility criteria. Disagreements were resolved by consensus.

### Data extraction

Two authors (NVF and NS) extracted all data, and disagreements were resolved by consensus. We used an excel spreadsheet to record data related to: (1) study characteristics (study design, level of evidence and country); (2) population characteristics (sample size, number of shoulder, mean age, female/male ratio, rate of upper limb dominance); (3) rotator cuff injury related parameters (grade of tear, size of tear, tendons involved, and acute vs. chronic tears); (4) rotator cuff repair (arthroscopic vs. open, type of fixation, any associated procedures and time of postoperative immobilization); (5) augmentation with MSCs or their derivatives (biologic product, source, volume, concentration, culture procedures and location of injection); and (6) baseline and post-intervention outcomes (PROMs, shoulder function, imaging and adverse events). The level of evidence (LoE) was registered as according to the 2011 version of the levels of evidence summary table of the Oxford Centre for Evidence-Based Medicine.^78^

### Risk of bias

The included studies comprise different study designs, and therefore the tools to judge the risk of bias varied according to the study design. Two authors (NVF and NS) individually judged the risk of bias of each study and any disagreements were resolved by consensus.

The risk of bias of the randomized studies was judged using the revised Cochrane risk-of-bias (RoB2) tool version 2.^97^ This tool assesses the following five bias domains: i) randomization, ii) deviations from intended interventions, iii) missing outcome data, iv) measurement of outcomes, and v) selection of the reported result. Each bias domain was judged as “low risk”, “some concerns” and “high risk”. The overall risk of bias judgment was based on the bias appraisal from the five domains.

The risk of bias of nonrandomized cohort studies was judged using the revised Risk of Bias in Nonrandomized Studies of Interventions (ROBINS-I) tool.^96^ This tool assesses seven bias domains: i) confounding, ii) selection of patients, iii) classification of interventions, iv) deviations from intended interventions, v) missing outcome data, vi) measurement of outcomes, viii) selection of the reported result. Each bias domain was judged as “low risk”, “moderate risk”, “serious risk”, “critical risk” and “no information”. The overall risk of bias judgement was based on the bias appraisal from the seven domains.

The risk of bias of other study designs was judged using the Risk of Bias Assessment tool for Nonrandomized Studies (RoBANS).^51^ This tool assesses six distinct bias domains: i) selection of participants, ii) confounding variables, iii) measurement of exposure, iv) blinding of outcome assessment, v) incomplete outcome data, vi) selective outcome reporting. Each bias domain was judged as “low risk”, “high risk” or “unclear”. No overall risk of bias judgement can be made with RoBANS tool, but due to the nature of these study designs, the overall risk of bias is judged as high risk.

### Data management and synthesis

Population characteristics are presented as pooled means and standard deviation (SD) weighted to the sample size (if continuous variables)^42^ or pooled rates (if dichotomous or categorical variables). When pain score using the numeric rating scale or the visual analog scale (VAS) was reported as 0-100, the data was transformed into 0-10 for uniformization. When pain was also reported as “at rest” and “at motion”, we collected both and prioritized “at motion” for the data synthesis.

A meta-analysis was not attempted due to the very low number of studies per comparison/outcome and the clinical heterogeneity across included studies. We collected the mean and SD for each outcome at baseline and at the several follow-up endpoints. When reported, the within-group and between-groups *P* values were also extracted from original studies. For the quantitative synthesis, we calculated the mean differences (with their respective SD and 95% confidence intervals [CIs]) for each outcome within each study at the latest follow-up endpoint. The SD of the within-group mean change was computed assuming a correlation coefficient of r = 0.5.^41^ We also calculated the between-group mean differences at the latest follow-up endpoint for studies that provided a MSCs and a control group without MSCs. The percentage of the mean change relative to the minimal clinically important difference (MCID) was calculated. For a reference value of MCID, previously published data^98^ for each outcome (determined for rotator cuff repair) were used: University of California at Los Angeles Shoulder Score with a MCID of 2.8; Constant score with a MCID of 10.9; American Shoulder and Elbow Surgeons score with a MCID of 17.8; Simple Shoulder Test with a MCID of 4.3; VAS pain with a MCID of −2.4. For range of motion (ROM) data, we could not find MCID for rotator cuff repair, so the MCID values for total shoulder arthroplasty are used instead: shoulder forward flexion with MCID of 11.7 degrees and external rotation with a MCID of 4.9 degrees.^94^ No MCID was available for shoulder strength. Other outcomes were synthetized as number and percentages (retears) or narratively (imaging). For retears, when reported in comparative studies, the between-group relative risk (RR) was also calculated.

## Results

### Study selection and characterization

**Figure 1:**
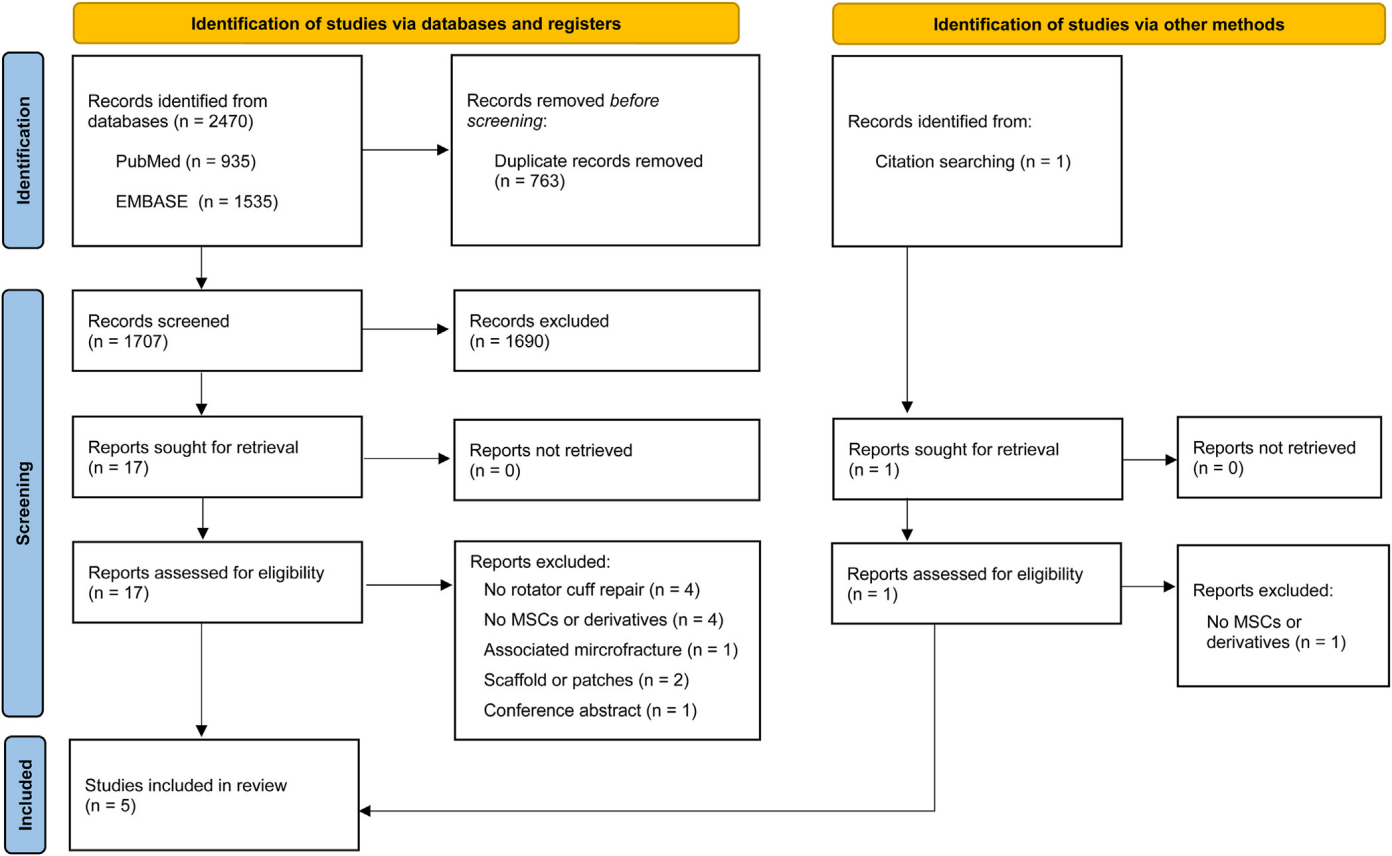
PRISMA flowchart for selection and inclusion of studies. *PRISMA*, Preferred Reporting Items for Systematic Reviews and Meta-Analyses; *MSCs*, mesenchymal stem cells

Database searches yielded 2470 results. Following the removal of duplicate records, there were 1707 unique records, of which 18 followed for full-text analysis. Hand searches yielded another study not identified in database searches. A total of 5 studies^27^^,^^36^^,^^39^^,^^53^^,^^84^ met the eligibility criteria and were included for qualitative analysis (Fig. 1).

There were three comparative studies (one LoE II,^84^ one LoE III,^53^ and another LoE IV^39^) and two case series (both LoE IV^27^^,^^36^). Three studies were performed in Europe, one in South America and another in the Asian continent.

### Risk of bias

**Figure 2:**
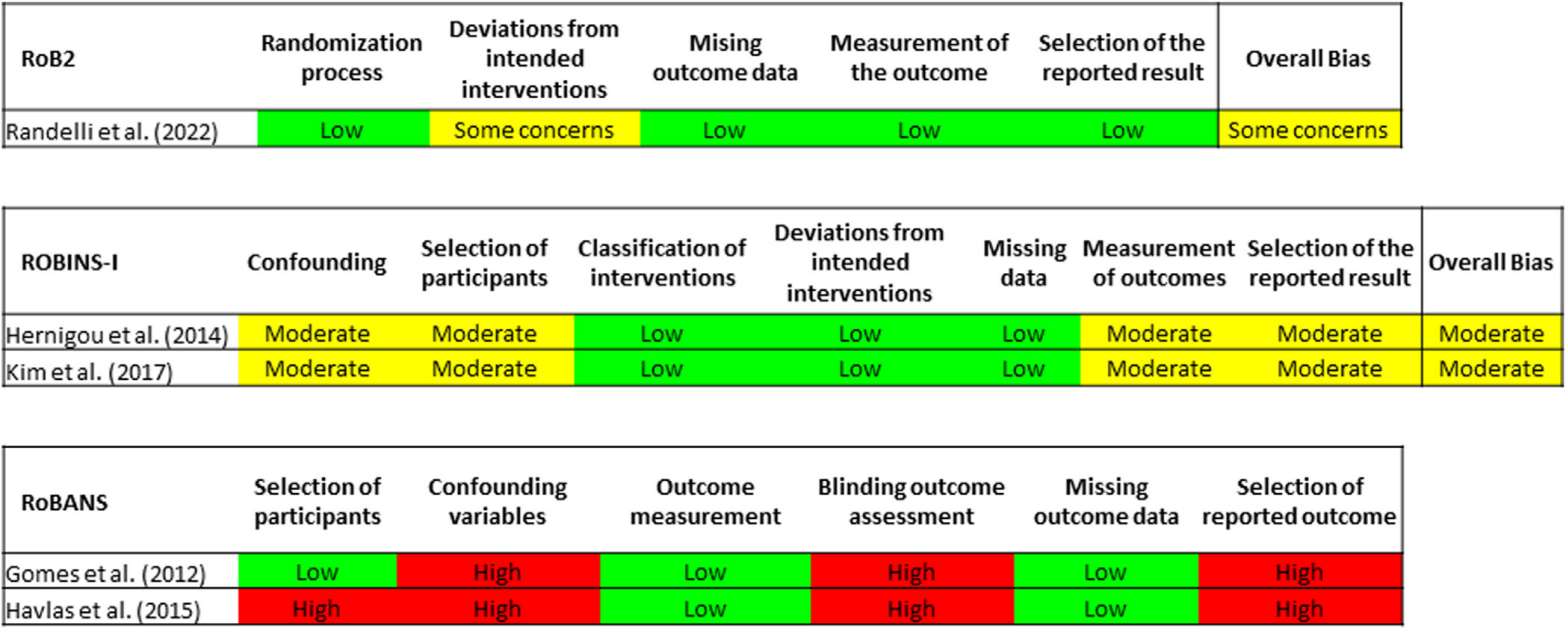
Results of judgment of risk of bias of included studies. *RoB2*, Cochrane risk-of-bias tool version 2; *ROBINS-I*, Risk of Bias in Nonrandomized Studies of Interventions; *RoBANS*, Risk of Bias Assessment tool for Nonrandomized Studies.

The randomized controlled trial^84^ was judged as “some concerns” as overall risk of bias due to some concerns related to interventions because both the surgeon performing the intervention, as well as the patients receiving the intervention, were aware of the intervention being received. Other bias domains were judged with low risk of bias (Fig. 2).

For the two nonrandomized cohort studies,^39^^,^^53^ both were judged as overall “moderate risk” of bias due to concerns in selection of participants, potentially noncontrolled confounders, nonblinding of outcome assessors and lack of published preintervention protocol. Other bias domains were judged with low risk of bias (Fig. 2).

The two-case series^27^^,^^36^ were judged with high risk of bias due to concerns in uncontrolled confounding variables (selection bias), lack of blinding of outcome assessor (detection bias) and lack of published preintervention protocol (selective reporting bias). One study^36^ was also at high risk of selection bias due to nonconsecutive selection of participants (Fig. 2). We judged these two-case series with an overall “high risk” of bias.

### Population and rotator cuff injury characteristics

There were a total of 310 individuals (44% males) with a weighted mean age of 59.3 ± 0.9 years (Table I). The rotator cuff repair with MSCs groups comprised 161 individuals and the control groups with rotator cuff repair alone included 149 individuals. All rotator cuff injuries were unilateral with no study including bilateral rotator cuff repairs. The sample sizes were mostly small, ranging from 8 to 81 participants per group.

**Table I:** Study and population characteristics.

Reference	Study design	LoE	Country	Group	N	Age	M:F	Upper limb dominance	Grade and type of tear	Size∗	Tendons involved
Gomes et al (2012)^27^	Case series	IV	Brazil	RCR + MSCs	14	59.2	5:9	NR	Grade II or higher (Goutallier): 10 patients	NR	3 tendons (n = 5) 2 tendons (n = 8) 1 tendon (n = 1)
Hernigou et al (2014)^39^†	Case-control	IV	France	RCR + MSCs	45	NR	NR	NR	Most had had grade 0 or grade 1 (Goutallier) [groups matched]	2.3 ± 0.5 cm	Supraspinatus
				RCR alone	45	61 (49–71)	28:25	26 (58%) as dominant		2.2 ± 0.5 cm	
Havlas et al (2015)^36^	Case series	IV	Czech Republic	RCR + MSCs	8	NR	NR	NR	NR	NR	Supraspinatus or infraspinatus
Kim et al (2017)^53^	Cohort clinical study	III	South Korea	RCR + MSCs	71	59.5 ± 3.6 (41-77)	30:42	34 right/38 left	NR	Medium (n = 39) Large (n = 26) Massive (n = 7)	Supraspinatus, infraspinatus, or subscapularis
				RCR alone	81	58.4 ± 3.1 (42-74)	32:49	39 right/42 left	NR	Medium (n = 41) Large (n = 33) Massive (n = 7)	
Randelli et al (2022)^84^‡	RCT	II	Italy	RCR + MSCs	23	58.4 ± 7.8	11:12	21 right/2 left	*SCOI classification* : C1 (n = 8), C2 (n = 10) and C3 (n = 5)§ *Tear shape*: Crescent (n = 16), U-shaped (n = 1), L-shaped (n = 4) and Inverted L (n = 2)	16.4 ± 6.8 mm (AP) 16.9 ± 12.1 mm (ML)	Supraspinatus and infraspinatus
				RCR alone	23	59.4 ± 6.3	8:15	18 right/5 left	*SCOI classification*: C1 (n = 11), C2 (n = 7) and C3 (n = 5) *Tear shape*: Crescent (n = 12), U-shaped (n = 3), L-shaped (n = 3) and Inverted L (n = 2)	13.57 ± 8.16 mm (AP) 16.10 ± 10.43 mm (ML)	Supraspinatus and infraspinatus

*RCR*, rotator cuff repair; *MSCs*, mesenchymal stem cells; *AP*, anteroposterior; *ML*, mediolateral; *RCT*, randomized controlled trial; *LoE*, level of evidence; *M:F*, male:female; *SCOI*, Southern California Orthopaedic Institute; *NR*, nonreported.

∗ Tear size classification: small (<1 cm), medium (≥1 cm to <3 cm), large (≥3 cm to <5 cm), or massive (≥5 cm).

† Some baseline data was missing, but groups were matched for baseline demographic data.

‡ Outcomes are related to 22 vs. 22 patients (1 drop-out in each group).

§ C1 - Small complete tear, pinhole sized; C2 - Moderate tear <2 cm of only one tendon without retraction; C3 - Large complete tear with an entire tendon with minimal retraction usually 3-4 cm.

The characteristics of rotator cuff tears were poorly reported. The type of rotator cuff tears ranged from small to massive tears with grades 0 to IV (Goutallier classification). Tears usually involved the supraspinatus and/or infraspinatus tendons, but the number of ruptured tendons was seldom reported. No study reported on the chronicity of rotator cuff tears.

### Intervention characteristics

Only one study^27^ used a mini-open approach with transosseous fixation; other studies employed an arthroscopic rotator cuff repair with single or double row fixation. All studies performed an associated acromioplasty in all patients^27^^,^^36^^,^^39^^,^^53^ or when patients presented a type 2 or 3 acromial morphology.^84^ One study^39^ also performed a coracoacromial ligament release in all patients. When reported, immobilization was one week with a sling,^39^ or four weeks with either a sling^27^^,^^84^ or a brace^36^ (Supplement 2).

Three studies^27^^,^^36^^,^^39^ used bone marrow MSCs (harvested from the iliac crest) and other two studies^53^^,^^84^ used adipose-derived MSCs (harvested from the abdomen or gluteal region). The adipose-derived MSCs were microfragmented before injection^84^ or coupled with fibrin glue during the injection procedure.^53^ Only two studies^36^^,^^53^ used a culturing procedure for MSCs expansion. The volume of MSCs injected varied substantially from 1.5 mL to 60-100 mL, with concentrations of MSCs ranging from 5.1 × 10^4^ to 3.81 × 10^8^ (Table II). The location of injection was usually at the site of repair, either at the footprint or at the bone-tendon junction.

**Table II:** Characteristics of mesenchymal stem cells and of the injection procedure.

Reference	Biologic product	Source	Culturing?	Volume (concentration)	Location of injection
Gomes et al (2012)^27^	BMMSCs	Autologous bone marrow (posterior iliac crest)	No	10 mL (3.81 × 10^8^)	Tendon and bone at the “footprint”
Hernigou et al (2014)^39^	BMMSCs	Autologous bone marrow (anterior iliac crest)	No	12 mL (5.1 ± 2.5 × 10^4^)	Tendon at the bone-tendon junction (8 ml), and in the bone at the footprint (4 ml)
Havlas et al (2015)^36^	BMMSCs	Autologous bone marrow (iliac crest)	Yes	1.5 mL (1.0 ± 0.45 × 10^7^)	Suture site
Kim et al (2017)^53^	AMSCs with fibrin glue	Autologous adipose tissue (gluteal region)	Yes	2 mL (4.46 × 10^6^)∗	Surface of the repaired tendon
Randelli et al (2022)^84^	Microfragmented AMSCs	Autologous adipose tissue (abdomen or gluteal region)	No	60-100 mL (NR)	At the repaired rotator cuff site

*BMMSCs*, bone marrow mesenchymal stem cells; *AMSCs*, adipose mesenchymal stem cells; *NR*, nonreported.

∗ The 2 ml contain MSCs gelled in fibrin glue.

### Imaging and surgical outcomes

All five studies reported tendon healing as evaluated with imaging procedures (Supplement 3). The two case series reported magnetic resonance imaging (MRI) tendon healing in all (at 6 months)^36^ or more than half of patients as shown by low signal intensity areas along the tendon (at 12 months).^27^ The three comparative studies reported tendon healing using different methods. Hernigou et al^39^ using ultrasound evaluation, reported that healing at 1-year follow-up was prominent or total more frequently in the MSCs group as compared with the control group. Kim et al^53^ using MRI, reported a higher rate of tendon healing in the MSCs group (84.7%) as compared to the control group (70.4%) at around 14 months follow-up. Lastly, Randelli et al^84^ also using MRI reported slightly higher rates of Sugaya tendon integrity type I and II in the MSCs group (68%) as compared to the control group (57%) at 18 months follow-up. Randelli et al^84^ also reported on muscle atrophy (Warner grading) and fatty degeneration (Fuchs score) at 18 months follow-up. Patients at the MSCs group displayed similar rates of none or mild muscle atrophy (82% vs. 82%) and fatty infiltration (10% vs. 9%)

Retears ranged from 0% to 15.3% in the rotator cuff repair with MSCs groups and from 17% to 56% in the rotator cuff repair alone groups. At mid-term follow-up one study^84^ showed that although the rotator cuff repair with MSCs group displayed lower retear rates than the control group with rotator cuff repair alone (9% vs. 17%), there was no significant protective effect in the RR (Table III). However, another study^53^ showed a protective effect with a reduction in risk of 48% (RR = 0.52, 95% CI 0.27-0.98) at mid-term follow-up with a lower rate of retear in the MSCs group (15.3% vs. 29.6%). At the long term (10 years),^39^ there was also a significant protective effect with a reduction in risk of 76% (RR = 0.24, 95% CI 0.11-0.53), representing a substantially lower retear rate in the rotator cuff repair with MSCs group (13%) as compared to the group with rotator cuff repair alone (56%).

**Table III:** Characteristics of mesenchymal stem cells and of the injection procedure.

Reference	Follow-up	Retears, n (%)		RR (95% CI)	Adverse events, number of patients (%)		RR (95% CI)
		RCR + MSCs	RCR alone		RCR + MSCs	RCR alone	
Gomes et al (2012)^27^	12 mo.	0 (0%)	NA	NA	NR	NA	NA
Hernigou et al (2014)^39^	10 yrs	6 (13%)	25 (56%)	0.24 (0.11-0.53)∗	NR	NR	NR
Havlas et al (2015)^36^	6 mo	0 (0%)	NA	NA	0 (0%)	NA	NA
Kim et al (2017)^53^	MSCs: 14.4 mo. (12-21) RCR: 14.0 mo. (12-22)	11 (15.3%)	24 (29.6%)	0.52 (0.27-0.98)∗	NR	NR	NR
Randelli et al (2022)^84^	18 mo.	2 (9%)	4 (17%)	0.52 (0.11-2.57)	11 (48%)	7 (30%)	1.57 (0.74-3.33)

*RCR*, rotator cuff repair; *MSCs*, mesenchymal stem cells; *mo*, months; *RR*, relative risk; *CI*, confidence interval; *NA*, nonapplicable; *NR*, nonreported

∗ Significant protective effect towards RCR + MSCs group.

One study (case series)^27^ that although not finding any retears, reported failure in one patient (7%) that presented recurrent pain and loss of strength, requiring a new surgical procedure.

### PROMs, shoulder ROM, and strength

The rotator cuff repair augmented with MSCs groups showed significant improvement in the University of California at Los Angeles Shoulder Score, Constant, American Shoulder and Elbow Surgeons, Simple Shoulder Test, and VAS scores at the latest follow-up. The MCID was achieved for all PROMs, ranging from a minimum of 135% to a maximum of 679%. Shoulder ROM and strength were significantly improved for forward flexion and external rotation, but not for internal rotation (Table IV). Supplement 4 details the full outcome reporting for each study and outcome at all follow-up endpoints.

**Table IV:** Improvement in PROMs, shoulder ROM and strength of MSCs groups.

Outcome	Follow-up	Baseline	Post-op (6 m)	Post-op (12 m)	Post-op (≥24 m)	MD ± SD (95% CI)∗	Within-group *P* value∗†	MCID (%)∗
UCLA (2-35)								
Gomes et al (2012)^27^	12 mo.	12 ± 3	---	31 ± 3.2	---	19.0 ± 3.1 (17.4-20.6)	NR	679
Havlas et al (2015)^36^	6 wks, 3 and 6 mo.	18.4 ± 6.4	32.0 ± 2.4	---	---	13.6 ± 5.6 (9.7-17.5)	NR	486
Kim et al (2017)^53^	31.2 ± 4.2 mo.	23.9 ± 5.4	---	---	30.4 ± 5.5	6.5 ± 5.5 (5.2-7.8)	*P* < .001	232
Constant score (0-100)								
Havlas et al (2015)^36^	6 wks, 3 and 6 mo.	40.9 ± 21.0	84.4 ± 4.7	---	---	43.5 ± 19.1 (30.2-56.7)	NR	399
Kim et al (2017)^53^	31.2 ± 4.2 mo.	65.5 ± 15.2	---	---	80.2 ± 15.4	14.7 ± 15.3 (11.2-18.2)	*P*<.001	135
Randelli et al (2022)^84^	3, 6, 12, 18 and 24 mo.	49.3 ± 15.4	82.7 ± 7.0	85.9 ± 6.3	86.3 ± 5.3	37.0 ± 13.6 (31.4-42.5)	*P* < .001	339
ASES (0-100)								
Randelli et al (2022)^84^	3, 6, 12, 18 and 24 mo.	48.0 ± 20.3	93.99 ± 6.94	95.76 ± 8.65	98.33 ± 4.66	50.3 ± 18.4 (42.8-57.8)	*P* < .001	283
SST (0-12)								
Randelli et al (2022)^84^	3, 6, 12, 18 and 24 mo.	5.22 ± 2.73	11.70 ± 0.47	11.77 ± 0.61	11.86 ± 0.35	6.6 ± 2.6 (5.6-7.7)	*P* < .001	154
VAS (10-0)								
Havlas et al (2015)^36^	6 wks, 3 and 6 mo.	5.3 ± 1.6	0.0 ± 0.0	---	---	−5.3 ± 1.6 (−6.4-−4.2)	NR	221
Kim et al (2017)^53^	31.2 ± 4.2 mo.	6.0 ± 1.6	---	---	2.3 ± 1.2	−3.7 ± 1.4 (−4.0-−3.4)	*P* < .001	154
Randelli et al (2022)^84^	3, 6, 12, 18 and 24 mo.	5.2 ± 2.2	0.5 ± 0.7	0.6 ± 1.5	0.2 ± 0.6	−5.0 ± 2.0 (−5.8-−4.2)	*P* < .001	208
Forward flexion (°)								
Kim et al (2017)^53^	31.2 ± 4.2 mo.	145.4 ± 20.4	---	---	153.2 ± 25.6	7.8 ± 23.4 (2.7-12.9)	*P* = .037	67
External rotation (°)								
Kim et al (2017)^53^	31.2 ± 4.2 mo.	51.9 ± 20.1	---	---	64.3 ± 24.1	12.4 ± 22.6 (7.2-17.6)	*P* = .034	253
Internal rotation‡								
Kim et al (2017)^53^	31.2 ± 4.2 mo.	T 10.9	---	---	T 10.6	- T 0.3	*P* = .784	---
Forward flexion (Kg)§								
Randelli et al (2022)^84^	3, 6, 12, 18 and 24 mo.	2.9 ± 2.0	4.9 ± 2.9	5.9 ± 3.1	6.2 ± 2.7	3.3 ± 2.4 (2.3-4.3)	*P* = .0006	---
External rotation (Kg)§								
Randelli et al (2022)^84^	3, 6, 12, 18 and 24 mo.	3.9 ± 1.9	6.1 ± 3.7	6.1 ± 3.7	7.2 ± 2.6	3.4 ± 2.3 (2.4-4.3)	*P* < .001	---

*UCLA*, University of California at Los Angeles Shoulder Score; *ASES*, American Shoulder and Elbow Surgeons score; *SST*, simple shoulder test; *VAS*, visual analog scale; *MD*, mean difference; *SD*, standard deviation; *CI*, confidence interval; *MCID*, minimal clinically important difference; *mo/m*, months; *wks*, weeks; *MSCs*, mesenchymal stem cells; *PROMs*, patient reported outcome measures; *ROM*, range of motion

∗ As related to the last follow-up.

† As reported in the original study.

‡ Internal rotation at the back was measured by the vertebral level reached by the tip of the thumb.

§ Isometric strength measured with a dynamometer.

When comparing the groups with rotator cuff repair augmented with MSCs against the control groups with rotator cuff repair alone, there were no significant differences across all outcomes at the latest follow-up (Table V). The MCIDs were also comparable between groups across outcomes.

**Table V:** Improvement in PROMs, shoulder ROM and strength between groups of comparative studies.

Outcome	Follow-up	Within-group MD ± SD (95% CI)∗		Between-group MD (95% CI)∗	Between-group *P* value†	MCID (%)	
		RCR + MSCs	RCR alone			RCR + MSCs	RCR alone
UCLA (2-35)							
Kim et al (2017)^53^	31.2 ± 4.2 mo.	6.5 ± 5.5 (5.2-7.8)	7.1 ± 5.5 (5.9-8.3)	−0.6 (−2.4 to 1.2)	*P* = .314	232	253
Constant score (0-100)							
Kim et al (2017)^53^	31.2 ± 4.2 mo.	14.7 ± 15.3 (11.2-18.2)	16.9 ± 15.7 (13.5-20.3)	−2.2 (−6.8 to 3.0)	*P* = .754	135	155
Randelli et al (2022)^84^	3, 6, 12, 18 and 24 mo.	37.0 ± 13.6 (31.4-42.5)	27.2 ± 16.3 (20.5-33.9)	9.8 (1.1-18.5)	*P* = .563	339	250
ASES (0-100)							
Randelli et al (2022)^84^	3, 6, 12, 18 and 24 mo.	50.3 ± 18.4 (42.8-57.8)	43.6 ± 14.5 (37.7-49.5)	6.7 (−2.9 to 16.3)	*P* = .198	283	244
SST (0-12)							
Randelli et al (2022)^84^	3, 6, 12, 18 and 24 mo.	6.6 ± 2.6 (5.6-7.7)	5.0 ± 2.7 (3.9-6.1)	1.6 (0.1-3.1)	*P* = .973	154	116
VAS (10-0)							
Kim et al (2017)^53^	31.2 ± 4.2 mo.	−3.7 ± 1.4 (−4.0 to −3.4)	−4.4 ± 1.6 (−4.7 to −4.1)	−0.7 (−1.2 to −0.2)	*P* = .872	154	183
Randelli et al (2022)^84^	3, 6, 12, 18 and 24 mo.	−5.0 ± 2.0 (−5.8 to −4.2)	−4.1 ± 2.4 (−5.1 to −3.1)	0.9 (−0.4 to 2.2)	*P* = .815	208	171
Forward flexion (°)							
Kim et al (2017)^53^	31.2 ± 4.2 mo.	7.8 ± 23.4 (2.7-12.9)	11.2 ± 23.5 (5.8-16.6)	−3.4 (−10.9 to 4.1)	*P* = .452	67	96
External rotation (°)							
Kim et al (2017)^53^	31.2 ± 4.2 mo.	12.4 ± 22.6 (7.2-17.6)	13.4 ± 20.9 (8.8-18.0)	−1.0 (−7.9 to 5.9)	*P* = .874	253	273
Internal rotation‡							
Kim et al (2017)^53^	31.2 ± 4.2 mo.	- T 0.3	- T 0.9	T 0.6	*P* = .206	---	---
Forward flexion (Kg)§							
Randelli et al (2022)^84^	3. 6. 12. 18 and 24 mo.	3.3 ± 2.4 (2.3-4.3)	1.5 ± 2.2 (0.6-2.5)	1.8 (0.7-3.1)	*P* = .128	---	---
External rotation (Kg)§							
Randelli et al (2022)^84^	3. 6. 12. 18 and 24 mo.	3.4 ± 2.3 (2.4-4.3)	2.5 ± 2.5 (1.5-3.6)	0.9 (−0.5-2.3)	*P* = .660	---	---

*RCR*, rotator cuff repair; *MSCs*, mesenchymal stem cells; *UCLA*, University of California at Los Angeles Shoulder Score; *ASES*, American Shoulder and Elbow Surgeons score; *SST*, simple shoulder test; *VAS*, visual analog scale; *MD*, mean difference; *SD*, standard deviation; *CI*, confidence interval; *MCID*, minimal clinically important difference; *mo/m*, months; *wks*, weeks; *PROMs*, patient reported outcome measures; *ROM*, range of motion.

∗ As related to the latest follow-up endpoint.

† As reported in the original study.

‡ Internal rotation at the back was measured by the vertebral level reached by the tip of the thumb.

§ Isometric strength measured with a dynamometer.

### Safety and adverse events

Two studies^36^^,^^84^ reported the occurrence of adverse events (Table III). One case series^36^ reported no adverse events. A randomized controlled trial^84^ reported 11 patients (48%) with 14 adverse events in the MSCs group against 7 patients (30%) with 11 adverse events in the control group; however, the RR was not significantly increased in the MSCs group (RR = 1.57, 95% CI 0.74-3.33), as well as all adverse events were resolved and there were no differences between the groups in regards to duration of recovery, severity of adverse events and relation of adverse events to the treatment.

## Discussion

The main finding of this systematic review is that there is no benefit in augmenting the rotator cuff repair with MSCs for clinical and functional benefit when comparing against an isolated rotator cuff repair. However, the aim of augmenting the repair with MSCs is not only to improve the clinical and functional outcomes, which is already achieved with rotator cuff repair alone,^15^^,^^24^^,^^46^^,^^58^^,^^64^^,^^82^^,^^87^^,^^89^^,^^92^^,^^118^ but to improve biological healing of the repaired rotator cuff and reduce the retear rates which remain unsatisfactorily high after isolated arthroscopic rotator cuff repair.^10^^,^^24^^,^^26^^,^^44^^,^^82^^,^^92^^,^^100^^,^^120^ The augmentation of the repair with MSCs resulted in a significant protective effect of 48% and 74% reduction in the risk of retearing the repaired rotator cuff at the mid-term^53^ and long-term^39^ follow-up. Tendon healing was also systematically better in the MSCs augmentation groups than in the isolated rotator cuff repair groups. The number and concentration of progenitor cells injected play crucial a role in improving repair healing and protecting against retear, with better outcomes when injections contained greater concentration of MSCs per cubic centimeter.^39^

The literature on this topic is still preliminary and these results should be interpreted with caution. There are only a handful of clinical human studies available, with only three comparison studies, and the quality and certainty of evidence at this moment is also still very limited. The available studies are also limited due to several concerns in risk of bias (often related to the nature of study design) and to the small sample sizes which are prone to type II error. Meta-analysis was not feasible, which precluded us from reliably appraising the certainty of evidence (Grading of Recommendations Assessment, Development and Evaluation); however, due to the nature of study designs, risk of bias, inconsistency and imprecision, the certainty of evidence is still very low, which reflects in weak recommendations with considerable degrees of uncertainty. Further well-design and high-powered studies are clearly warranted to draw stronger conclusions.

The bulk of evidence of MSCs to potentiate tendon healing during rotator cuff repair comes from preclinical animal studies.^18^^,^^29^, ^30^, ^31^, ^32^^-^^33^^,^^50^^,^^76^^,^^86^^,^^102^^,^^106^ Adding MSCs to rotator cuff repair improves the quality of tissue surrounding the area under repair by improving biomechanical properties of the repaired rotator cuff, and enhancing biological healing^61^^,^^71^^,^^110^; however, these improvements are not seen across all preclinical studies and were often only significant when the MSCs were embedded into scaffold.^71^ There are also a few clinical studies that report the results of injecting MSCs into the injured rotator cuff when not associated to a surgical procedure.^12^^,^^45^^,^^47^^,^^48^ These studies show clinical and functional improvements after the injection of MSCs into the rotator cuff without relevant adverse events up to the 2-year follow-up. However, MSCs injections without rotator cuff repair are only indicated for patients with partial-thickness tears, and are thus limited in their scope.

### Harvesting sources of MSCs

The MSCs sources used in the included studies of our systematic review were either the bone marrow or the adipose tissue, which are the most commonly used also for other musculoskeletal conditions.^11^^,^^81^^,^^103^^,^^104^^,^^109^^,^^116^ Although the harvesting location was similar for either source, the preparations were heterogenous across the studies (specially for the adipose-derived MSCs) which precludes direct comparison on preparation procedures. The currently available evidence does not allow determining which would be the best source for MSCs for the augmentation of rotator cuff repair and further studies are required to investigate if there is any effect regarding the choice of source.

There are other relevant harvesting sources containing MSCs, including the bursa,^4^^,^^23^^,^^54^^,^^55^^,^^57^^,^^60^^,^^67^, ^68^, ^69^^-^^70^^,^^72^^,^^73^^,^^101^^,^^115^ the tendon,^7^^,^^19^^,^^93^^,^^95^^,^^101^^,^^105^^,^^107^^,^^125^ the umbilical cord^56^^,^^80^^,^^83^^,^^119^ and the synovial.^5^ The subacromial bursa has been recently widely investigated as an easily accessible and convenient source to harvest MSCs during the rotator cuff repair. Despite recent research on the subacromial bursa as a potential useful source of MSCs for rotator cuff repair, there is still very limited clinical evidence and not study injected bursa-derived MSCs as augmentation for rotator cuff repair.

### Future directions

There a clear need for further well-designed and high-powered studies that compare the rotator cuff repair with and without the augmentation with MSCs to drawn stronger and more definitive conclusions. The body of evidence is currently only composed by three comparative studies and two case series, all judged with having risk of bias in one or more bias domains, which is insufficient and provides very-low certainty when drawing recommendations.

Although the potential of MSCs has not yet been fully clarified, it has been proposed that beyond its differentiation capacity and cell-cell interaction, the secretion of a wide range of biological factors, known as secretome, could be the main reason of their regenerative capacity in the lesion sites.^3^^,^^90^ The MSCs’ secretome can act as a regulator/modulator of different cellular processes such as proliferation, differentiation, communication and migration.^16^^,^^88^ The secretome has shown very promising results in preclinical studies,^17^^,^^25^^,^^34^^,^^37^^,^^43^^,^^52^^,^^63^^,^^85^^,^^90^^,^^91^^,^^108^^,^^110^, ^111^, ^112^, ^113^^-^^113^^,^^121^, ^122^, ^123^^-^^124^ but remains to be investigated in association to rotator cuff repair in humans.

The available studies are still heterogenous on the sources of MSCs, isolation and preparation procedures, and culturing/expansion of MSCs. Future studies should aim to standardize these steps according to international consensus guidelines.^21^^,^^66^^,^^74^ The available studies focus only on MSCs harvested from the bone marrow and adipose tissue, and no conclusion can be made on which is the best source, requiring more studies to allow a quantitative comparison using meta-analytic procedures. The bursa has been emerging as a potential source of MSCs with special interest to the rotator cuff due to the accessibility to harvest during rotator cuff surgery and studies have shown that is reliable source of MSCs.^4^^,^^23^^,^^60^^,^^68^^,^^72^ However, we now need studies that apply bursa-derived MSCs to the repaired rotator cuff to investigate if this source yields comparable or superior effects than other commonly used sources.

### Limitations

Our systematic review has some limitations. We were not able to perform a meta-analysis due to the high clinical heterogeneity across the included studies (different sources and outcome measures) and to the very low number of available comparative studies. Although these factors precluded us from reliably pooling the results of these studies, we synthesized the within- and between-group results for each study and outcome, which provides an insight into patient improvement and group differences. The different study designs of included studies precluded us from performing a uniform analysis of risk of bias; we applied different risk of bias tools that were more adequate to each study design.

We planned (*a priori* protocol) to also analyze the derivates of MSCs, such as the secretome, as an augmentation too for rotator cuff repair. However, no such clinical study in humans is available in the literature and evidence analysis for secretome injections was not possible.

There was a case-control study^39^ that compared groups using a matched sample, but would be interesting to have also the results of the total unmatched sample to increase the power of analysis (especially the retar and heling rates which are more prone to type II error). Another study^53^ also presented a matched sample, but they provided also the total sample, which we used for synthesizing the data.

Another limitation of our systematic review is related to the use of an MCID of shoulder ROM that was based on a study on total shoulder arthroplasty,^94^ because there were no studies that calculated the MCID for patients undergoing rotator cuff repair. Patients indicated to total shoulder arthroplasty usually display more compromised shoulder ROM than patients indicated to a rotator cuff repair, and improvements in ROM are usually greater after a total shoulder arthroplasty than after a rotator cuff repair (especially in forward flexion), which may result in higher MCID cut-off; this fact may explain the low percentages of MCID achieved in both groups, which did not reach the MCID of 11.7 degrees.

## Conclusion

The currently available data on human clinical studies does not show any significant differences clinical and functional outcomes between rotator cuff repair with or without the augmentation of MSCs. However, there is a protective effect against retear when augmenting the rotator cuff repair with MSCs at mid-term and long-term follow-up. The literature on this topic is yet preliminary and the quality and certainty of evidence is still limited. There is a clear need for well-design and high-powered studies to draw stronger conclusions.

## Disclaimers:

Funding: No funding was disclosed by the authors.

Conflicts of interest: The authors, their immediate families, and any research foundation with which they are affiliated have not received any financial payments or other benefits from any commercial entity related to the subject of this article.
