# Supplementary material for: The role of injections of mesenchymal stem cells as an augmentation tool in rotator cuff repair: a systematic review
Source: JSES Rev Rep Tech. 2025 Jan 13;5(2):231–42. doi: 10.1016/j.xrrt.2024.12.003 (PMC12047555; doi:10.1016/j.xrrt.2024.12.003)
Supplement: Supplement 1 [file mmc1.docx]

**Supplement 1 –** Database search strategy.

| **Database** | **Search strategy** |
| --- | --- |
| **PubMed** | ((("Mesenchymal Stem Cell Transplantation"[MeSH] OR "Mesenchymal Stromal Cells"[MeSH] OR (mesenchym* OR stem cell* OR secretome OR "extracellular vesicle") OR ("bone marrow" OR "adipose stem" OR "adipose mesenchymal stem" OR "adipose-derived" OR "adipose-derived mesenchymal stem" OR "stromal vascular fraction")) AND ("rotator cuff"[tiab] OR shoulder[tiab]) |
| **EMBASE** | ('mesenchymal stem cell transplantation'/exp/mj OR 'mesenchymal stromal cells'/exp/mj OR ((mesenchym* OR stem) AND cell*) OR secretome OR 'extracellular vesicle' OR 'bone marrow' OR 'adipose stem' OR 'adipose mesenchymal stem' OR 'adipose-derived' OR 'adipose-derived mesenchymal stem' OR 'stromal vascular fraction') AND ('rotator cuff':ab,ti OR shoulder:ab,ti) |
