# Supplementary material for: The role of injections of mesenchymal stem cells as an augmentation tool in rotator cuff repair: a systematic review
Source: JSES Rev Rep Tech. 2025 Jan 13;5(2):231–42. doi: 10.1016/j.xrrt.2024.12.003 (PMC12047555; doi:10.1016/j.xrrt.2024.12.003)
Supplement: Supplement 2 [file mmc2.docx]

**Supplement 2 –** Characteristics of rotator cuff repair procedure.

| **Reference** | **Surgical approach** | **Fixation** | **Associated procedures** | **Duration of post-op immobilization** |
| --- | --- | --- | --- | --- |
| Gomes et al. (2012) | Mini-open | Transosseous fixation | Acromioplasty (100%) | 4 weeks (sling) |
| Hernigou et al. (2014) | Arthroscopic | Single row | Acromioplasty (100%) and CAL release (100%) | 1 week (sling) |
| Havlas et al. (2015) | Arthroscopic | NR | Acromioplasty (100%) | 4 weeks (brace) |
| Kim et al. (2017) | Arthroscopic | Double row | Acromioplasty (100%) | NR |
| Randelli et al. (2022) | Arthroscopic | Single row | Acromioplasty | 4 weeks (sling) |

**Legend**: CAL - coracoacromial ligament; Post-op – Post-operative; NR – Non-reported.
