# Supplementary material for: The role of injections of mesenchymal stem cells as an augmentation tool in rotator cuff repair: a systematic review
Source: JSES Rev Rep Tech. 2025 Jan 13;5(2):231–42. doi: 10.1016/j.xrrt.2024.12.003 (PMC12047555; doi:10.1016/j.xrrt.2024.12.003)
Supplement: Supplement 3 [file mmc3.docx]

**Supplement 3 –** Follow-up imaging outcomes.

| **Reference** | **Follow-up** | **Imaging procedure** | **Imaging outcomes** |
| --- | --- | --- | --- |
| Gomes et al. (2012) | 12 mo. | MRI | - Presence of low signal intensity areas along the supraspinatus tendon and distal muscle belly in 8 cases; - High-intensity blooming small round artifact at the bursal and tendon topography in 11 cases; - Formation of a high-signal intensity zone at the critical zone in 6 cases. |
| Hernigou et al. (2014) | 12 and 24 mo. | US | **Healing surface of the footprint***  1-year: Healing was more frequently graded prominent, or total in the MSCs group as compared with the control group.  2 -years: sparse (6 cases) or moderate (4 cases) among the 10 failures, although there was no evidence of fluid signal density within the rotator cuff tendon indicating an absence of full-thickness tendon gaps. |
| Havlas et al. (2015) | 6 mo. | MRI | Fully healed and rebuilt rotator cuff attachment tissue in all 8 evaluated patients. |
| Kim et al. (2017) | **MSCs**: 13.9 mo. (12-21)  **RCR**: 14.3 mo. (12-17) | MRI | **RCR + MSCs**: rotator cuff tendon was completely healed in 61 of 72 patients (84.7%)  **RCR alone**: rotator cuff tendon was completely healed in 57 of 81 patients (70.4%) |
| Randelli et al. (2022) | 18 mo. | MRI | **RCR + MSCs**  - Sugaya tendon integrity type: 1 (n=4, 18%), 2 (n=11, 50%), 3 (n=3, 14%), 4 (n=4, 18%) and 5 (n=0, 0%) - Warner atrophy grade: 1 (n=6, 27%), 2 (n=12, 55%), 3 (n=3, 14%) and 4 (n=1, 5%) - Fuchs fatty degeneration score: 0 (n=5, 23%), 1 (n=9, 41%), 2 (n=6, 27%), 3 (n=1, 5%) and 4 (n=1, 5%)  **RCR alone**  - Sugaya tendon integrity type: 1 (n=8, 35%), 2 (n=5, 22%), 3 (n=3, 13%), 4 (n=3, 13%) and 5 (n=4, 17 %)  - Warner atrophy grade: 1 (n=7, 30%), 2 (n=12, 52%), 3 (n=3, 13%) and 4 (n=1, 4%)  - Fuchs fatty degeneration score: 0 (n=5, 22%), 1 (n=12, 52%), 2 (n=4, 17%), 3 (n=0, 0%) and 4 (n=2, 9%) |

**Legend**: RCR – Rotator cuff repair; MSCs – Mesenchymal stem cells; mo – months; MRI – Magnetic resonance imaging; US – Ultrasound; NA – Non-applicable.

* Absent (grade 0), sparse (grade 1), moderate (grade 2), prominent (grade 3), or total (grade 4)
