# Supplementary material for: The role of injections of mesenchymal stem cells as an augmentation tool in rotator cuff repair: a systematic review
Source: JSES Rev Rep Tech. 2025 Jan 13;5(2):231–42. doi: 10.1016/j.xrrt.2024.12.003 (PMC12047555; doi:10.1016/j.xrrt.2024.12.003)
Supplement: Supplement 4 [file mmc4.docx]

**Supplement 4** – Complete outcomes (PROMS, shoulder ROM and strength) for all studies and all endpoints.

| **Reference** | **Gomes et al. (2012)** | **Havlas et al. (2015)** | **Kim et al. (2017)** | | **Randelli et al. (2022)** | |
| --- | --- | --- | --- | --- | --- | --- |
| **Groups** | RCR + MSCs | RCR + MSCs | RCR + MSCs | RCR alone | RCR + MSCs | RCR alone |
| **Follow-up** | 12 mo. | 6 wks, 3 and 6 mo. | 31.2 ± 4.2 (24-56) | 27.9 ± 4.1 (24-38) | 3, 6, 12, 18 and 24 mo. | 3, 6, 12, 18 and 24 mo. |
| **UCLA - pre** | 12 ± 3 | 18.4 ± 6.4 | 23.9 ± 5.4 | 30.4 ± 5.5 | --- | --- |
| **UCLA - post** | 31 ± 3.2 | 6w: 20.4 ± 5.3  3m: 27.9 ± 4.9  6m: 32.0 ± 2.4 | 30.4 ± 5.5 | 31.3 ± 5.2 | --- | --- |
| **UCLA - P within** | NR | NR | P<0.001 | P<0.001 | --- | --- |
| **UCLA - P between** | --- | --- | P=0.314 | | --- | |
| **Constant - pre** | --- | 40.9 ± 21.0 | 65.5 ± 15.2 | 65.2 ± 16.1 | 49.31 ± 15.41 | 57.33 ± 18.41 |
| **Constant - post** | --- | 6w: 50.4 ± 17.8 3m: 67.3 ± 20.8 6m: 84.4 ± 4.7 | 80.2 ± 15.4 | 82.1 ± 15.2 | 3m: 69.66 ± 12.29 6m: 82.72 ± 7.00  12m: 85.86 ± 6.25 18m: 85.24 ± 6.89 24m: 86.28 ± 5.34 | 3m: 60.40 ± 17.54 6m: 76.66 ± 10.77  12m: 81.79 ± 7.60  18m: 83.34 ± 7.47 24m: 84.54 ± 5.93 |
| **Constant - P within** | --- | NR | P<0.001 | P<0.001 | P<0.001 | P<0.001 |
| **Constant - P between** | --- | --- | P=0.754 | | 6m: P=0.0050; 24m: P=0.5632 | |
| **VAS - pre** | --- | 5.3 ± 1.6 | 2.0 ± 0.9 (at rest) 6.0 ± 1.6 (at motion) | 2.2 ± 1.0 (at rest) 6.6 ± 1.8 (at motion) | 5.20 ± 2.24 | 4.39 ± 2.59 |
| **VAS - post** | --- | 6w: 1.4 ± 0.8 3m: 0.6 ± 0.5 6m: 0.0 ± 0.0 | 0.3 ± 0.7 (at rest) 2.3 ± 1.2 (at motion) | 0.3 ± 0.6 (at rest) 2.2 ± 1.0 (at motion) | 3m: 0.64 ± 1.11  6m: 0.51 ± 0.65 12m: 0.59 ± 1.50 18m: 0.27 ± 0.65 24m: 0.18 ± 0.61 | 3m: 1.20 ± 1.35 6m: 1.17 ± 1.72 12m: 0.77 ± 1.38 18m: 0.52 ± 1.06 24m: 0.25 ± 0.50 |
| **VAS - P within** | --- | NR | P<0.001 | P<0.001 | P<0.001 | P<0.001 |
| **VAS - P between** | --- | --- | P=0.214 (at rest); P=0.872 (at motion) | | 6m: P=0.2617; 24m: P=0.8152 | |
| **ASES - pre** | --- | --- | --- | --- | 48.04 ± 20.28 | 51.01 ± 16.71 |
| **ASES - post** | --- | --- | --- | --- | 3m: 81.74 ± 13.16 6m: 93.99 ± 6.94 12m: 95.76 ± 8.65 18m: 97.35 ± 5.36 24m: 98.33 ± 4.66 | 3m: 76.59 ± 16.40 6m: 85.14 ± 14.14 12m: 91.30 ± 10.17 18m: 93.48 ± 7.83 24m: 94.62 ± 8.51 |
| **ASES - P within** | --- | --- | --- | --- | P<0.001 | P<0.001 |
| **ASES - P between** | --- | --- | --- | | 6m: P=0.0265; 24m: P=0.1976 | |
| **SST - pre** | --- | --- | --- | --- | 5.22 ± 2.73 | 6.91 ± 2.83 |
| **SST - post** | --- | --- | --- | --- | 3m: 9.86 ± 2.25 6m: 11.70 ± 0.47 12m: 11.77 ± 0.61  18m: 11.68 ± 0.89 24m: 11.86 ± 0.35 | 3m: 7.65 ± 3.11  6m: 10.91 ± 1.47  12m: 11.43 ± 1.08 18m: 11.65 ± 0.65 24m: 11.91 ± 0.29 |
| **SST - P within** | --- | --- | --- | --- | P<0.001 | P<0.001 |
| **SST - P between** | --- | --- | --- | | 6m: P=0.0164; 24m: P=0.9733 | |
| **FF - pre** | --- | --- | 145.4 ± 20.4 | 144.1 ± 22.4 | --- | --- |
| **FF - post** | --- | --- | 153.2 ± 25.6 | 155.3 ± 24.5 | --- | --- |
| **FF - P within** | --- | --- | P=0.037 | P=0.023 | --- | --- |
| **FF - P between** | --- | --- | P=0.452 | | --- | |
| **ER - pre** | --- | --- | 51.9 ± 20.1 | 52.5 ± 19.2 | --- | --- |
| **ER - post** | --- | --- | 64.3 ± 24.1 | 65.9 ± 22.3 | --- | --- |
| **ER - P within** | --- | --- | P=0.034 | P<0.001 | --- | --- |
| **ER - P between** | --- | --- | P=0.874 | | --- | |
| **IR - pre** | --- | --- | T 10.9 | T 10.6 | --- | --- |
| **IR - post** | --- | --- | T 10.6 | T 9.7 | --- | --- |
| **IR - P within** | --- | --- | P=0.784 | P=0.418 | --- | --- |
| **IR - P between** | --- | --- | P=0.206 | | --- | |
| **FF (Kg) - pre** | --- | --- | --- | --- | 2.89 ± 2.00 | 3.63 ± 2.28 |
| **FF (Kg) - post** | --- | --- | --- | --- | 3m: 3.34 ± 2.16 6m: 4.89 ± 2.93 12m: 5.88 ± 3.06 18m: 5.62 ± 2.79 24m: 6.20 ± 2.65 | 3m: 2.78 ± 1.98 6m: 4.22 ± 2.24 12m: 4.80 ± 1.98 18m: 5.00 ± 2.35 24m: 5.17 ± 2.18 |
| **FF (Kg) - P within** | --- | --- | --- | --- | P=0.0006 | P<0.001 |
| **FF (Kg) - P between** | --- | --- | --- | | 6m: P=0.5010; 24m: P=0.128 | |
| **ER (Kg) - pre** | --- | --- | --- | --- | 3.85 ± 1.92 | 3.80 ± 1.85 |
| **ER (Kg) - post** | --- | --- | --- | --- | 3m: 4.62 ± 2.87 6m: 6.06 ± 3.68 12m: 6.06 ± 3.68 18m: 6.85 ± 2.87 24m: 7.23 ± 2.59 | 3m: 3.95 ± 2.10 6m: 5.11 ± 2.65 12m: 5.81 ± 2.60 18m: 6.20 ± 2.81 24m: 6.32 ± 2.90 |
| **ER (Kg) - P within** | --- | --- | --- | --- | P<0.001 | P<0.001 |
| **ER (Kg) - P between** | --- | --- | --- | | 6m: P=0.0077; 24m: P=0.6601 | |

**Legend**: RCR – Rotator cuff repair; MSCs – Mesenchymal stem cells; UCLA - University of California at Los Angeles Shoulder Score; ASES - American Shoulder and Elbow Surgeons score; SST - Simple Shoulder Test; VAS – Visual A---logue Scale; mo/m – months; wks – weeks.

**Note:** Hernigou et al. (2014) does not report any PROMS, neither shoulder ROM or strength.
